# 233. Osteomyelitis of the jaw: A retrospective analysis of clinical, microbiologic characteristics and antimicrobial treatment at a Tertiary Care Medical Center

**DOI:** 10.1093/ofid/ofab466.435

**Published:** 2021-12-04

**Authors:** Thinh Nguyen, Sudheer Surpure, Leonor Echevarria

**Affiliations:** 1 Banner Health, Phoenix, Arizona; 2 Grand Canyon Oral and Facial Surgery, Henderson, Nevada; 3 Albuquerque Veterans Affairs Medical Center, Albuquerque, New Mexico

## Abstract

**Background:**

Osteomyelitis of the jaw is a relatively rare entity in the post antibiotic era. The aim of this study is to describe clinical characteristics, microbiology and antibiotics use (oral vs intravenous) for treatment. We review 5 years of experience at Banner University Medical Center-Phoenix (BUMC-P) of proven cases of OM jaw by clinical, pathological, radiological criteria.

**Methods:**

Retrospective study of cases. From January 2011 to November 2015 ,157 cases of osteomyelitis of the jaw, we excluded cases of radiation therapy or neoplasia to the head and neck region, a history of antiresorptive medication use. A total of 34 patients with diagnosis of osteomyelitis of the jaw were reviewed. All patients met criteria for diagnosis of osteomyelitis and underwent surgical debridement and received antibiotics that included parenteral, orals and combined. We reviewed clinical, microbiology, antibiotic use. A successful outcome was defined as elimination of clinical symptoms, restoration of function and if available radiographic evidence of arrest and resolution of bony necrosis.

**Results:**

This retrospective study involved 34 patients. Most common organisms were oropharyngeal flora 22 samples (65%): streptococcus anginosus group. 4 samples grew unusual gram negative bacteria. 10 (29%) samples grew fungal species. Antimicrobial regimen was divided in: intravenous (n=14) (41.2%), oral (n=7) (20.6%) and combination intravenous followed by orals as follows: 13 (38.2 %).The average antibiotic duration was 8.1 + 4.7 weeks. We were able to follow up 30 patients, average follow up was 32.1-44.7 weeks. The overall success rate was (n=24) 80% with uneventful healing and. (n=6) (20%) treatment failure. There was more failure in the oral antibiotics group (n=3).

**Conclusion:**

This study is limited by small numbers. Surgery and cultures should guide treatment of osteomyelitis of the jaw. The use of oral antimicrobial therapy was associated to a higher likelihood of treatment failure. Although rarely linked as a cause of osteomyelitis, the authors think that the cultivation of candida spp should prompt appropriate coverage. More study is required to understand the efficacy of oral antimicrobial therapy in treating osteomyelitis of the jaw.

**Disclosures:**

**All Authors**: No reported disclosures

